# The impact of catch-up bivalent human papillomavirus vaccination on cervical screening outcomes: an observational study from the English HPV primary screening pilot

**DOI:** 10.1038/s41416-022-01791-w

**Published:** 2022-03-26

**Authors:** Matejka Rebolj, Francesca Pesola, Christopher Mathews, David Mesher, Kate Soldan, Henry Kitchener

**Affiliations:** 1 Cancer Prevention Group, School of Cancer & Pharmaceutical Sciences, Faculty of Life Sciences & Medicine, King’s College London, Great Maze Pond, London, SE1 9RT UK; 2 Cancer Prevention Trials Unit, School of Cancer & Pharmaceutical Sciences, Faculty of Life Sciences & Medicine, King’s College London, Great Maze Pond, London, SE1 9RT UK; 3 Blood Safety, Hepatitis, STI and HIV (BSHSH) Division, National Infection Service, Public Health England, 61 Colindale Avenue, London, NW9 5EQ UK; 4 Division of Cancer Sciences, University of Manchester, Oxford Road, Manchester, M13 9PL UK; 5 Present Address: Health and Lifestyle Research Unit, Wolfson Institute of Preventive Medicine, Queen Mary University of London, London, UK

**Keywords:** Population screening, Cancer prevention

## Abstract

**Background:**

In England, bivalent vaccination (Cervarix) against high-risk human papillomavirus (HR-HPV) genotypes 16/18 was offered in a population-based catch-up campaign in 2008–2010 to girls aged 14–17 years. These women are now entering the national cervical screening programme. We determined the impact of catch-up bivalent vaccination on their screening outcomes.

**Methods:**

We studied the overall and genotype-specific screening outcomes in 108,138 women aged 24–25 (offered vaccination) and 26–29 years (not offered vaccination) included in the English HPV screening pilot between 2013 and 2018.

**Results:**

At 24–25 years, the detection of high-grade cervical intraepithelial neoplasia (CIN2+) associated with HPV16/18 decreased from 3 to 1% (*p* < 0.001), with estimated vaccine effectiveness of 87% (95% CI: 82–91%). The detection of any CIN2+ halved from 6 to 3% (*p* < 0.001), with an estimated vaccine effectiveness of 72% (95% CI: 66–77%). The positive predictive value of a colposcopy for CIN2+ decreased for both low-grade (*p* < 0.001) and high-grade (*p* = 0.02) abnormalities on triage cytology. The decreases in screen-detected abnormalities at age 26-29 were of a substantially smaller magnitude.

**Conclusions:**

These data confirm high effectiveness of bivalent HPV vaccination delivered through a population-based catch-up campaign in England. These findings add to the rationale for extending screening intervals for vaccinated cohorts.

## Introduction

Approximately 70% [1] of cervical cancers (and >80% [2] in the UK) are caused by persistent infection with high-risk human papillomavirus (HR-HPV) genotypes 16 and 18. Vaccination targeting these two genotypes is highly effective in the prevention of persistent infections and associated cervical abnormalities including high-grade cervical intraepithelial lesions (CIN) and cancer [3], particularly when administered before sexual debut. The vaccine is still effective when administered later in adolescence and beyond, with somewhat reduced effectiveness due to infections acquired before vaccination [4, 5]. Since September 2008, the HPV vaccine has been routinely administered throughout England, through the national adolescent HPV vaccination programme to girls aged 12–13 years born on or after 1 September 1995 (Supplementary Information, Fig. S1). The delivery of this programme has been largely school-based. A catch-up campaign was also run during the period of 2008–2010, targeting girls aged 14–17 years born between 1 September 1990 and 31 August 1995. The catch-up campaign was provided by a mixture of general practice and school-based delivery. In September 2012, the programme changed from using the bivalent vaccine (Cervarix; GSK, Brentford, UK) to using the quadrivalent vaccine (Gardasil; Merck, Kenilworth, NJ), which also provides protection against genital warts [6]. Both vaccines have been shown to provide some protection against certain other HR-HPV genotypes such as 31 and 45 through cross-protection. The recommended vaccination schedule was originally three doses and changed to two doses in September 2014. All doses are administered free of charge. Vaccination uptake has been consistently high at 80–90% for the routine programme, while the catch-up campaign reached 40–75% of girls, depending on the birth cohort [7].

The National Health Service Cervical Screening Programme (NHS CSP) invites women for their first cervical screening 6 months before their 25th birthday. This means that the first cohorts in the catch-up campaign eligible for vaccination were invited for screening in March 2015. In 2013, a national pilot of primary screening with HR-HPV testing, followed by cytology if HR-HPV positive, was launched in six CSP laboratories. The pilot included ~1.3 million women whose primary screening test was either a HR-HPV test or liquid-based cytology (LBC) [8]. In routine screening, the pilot confirmed that primary screening with HR-HPV testing is more effective than primary screening with LBC in detecting underlying high-grade CIN (CIN2+ and CIN3+), reducing the development of invasive cervical cancer, and allowing longer screening intervals for women who test HR-HPV negative [8]. These data represent the most complete documentation of CSP outcomes in both unvaccinated women and the oldest women who were eligible for catch-up vaccination and who received cervical screening with HR-HPV as the primary test at age 25 years.

The aim of this study was to determine the impact of the catch-up vaccination programme with the bivalent vaccine administered at the age of 14 years and older on screening outcomes in the CSP pilot of HR-HPV testing for primary cervical screening. We report (a) the prevalence of all HR-HPV and, for a subset where this is known, of genotype-specific 16/18 infections, (b) the proportion of women referred to colposcopy, (c) the proportion of women diagnosed with CIN1, CIN2+, CIN3+, and cervical cancer detected by screening, and (d) the positive predictive value (PPV) of colposcopy for CIN2+.

## Methods

The pilot has been described in detail [8–10]. In summary, the pilot was launched in April 2013 and was embedded in the English CSP, which routinely recalls women aged between 25 and 49 years every 3 years (the first invitation is sent at 24.5 years) and women between 50 and 64 years every 5 years. The laboratories used either ThinPrep (Hologic, Marlborough, MA) or SurePath (BD, Sparks, MD) systems for LBC and APTIMA (Hologic, Manchester, UK), cobas 4800/6800 (Roche, Rotkreuz, Switzerland, or Branchburg, NJ) or RealTime (Abbott, Wiesbaden, Germany) HR-HPV assays. The cobas and RealTime assays were used in four laboratories. These two assays allow partial HR-HPV genotyping by detecting HPV 16 and HPV 18 DNA separately and reporting the detection of the DNA of the “other” 12 HR-HPV genotypes (31, 33, 35, 39, 45, 51, 52, 56, 58, 59, 66, 68) in combination. APTIMA, used in two pilot laboratories, reports the detection of mRNA from the same 14 HR-HPV genotypes in combination.

Women screened with HR-HPV tests were returned to routine recall if they tested negative. Samples found to be HR-HPV positive were sent for cytology triage. Women with non-negative cytology were referred to colposcopy at baseline. Non-negative cytology was defined as borderline changes in squamous or endocervical cells or worse, which is approximately consistent with atypical squamous or glandular cells of undetermined significance or worse in the Bethesda 2014 classification. Women with negative cytology had early recalls at 12 and 24 months, although those results could not yet be included in the analysis owing to few women who had undergone early recall after having been first screened in 2016 or later.

Data from the first (prevalence) round of primary screening by HR-HPV testing were retrieved from the laboratory information systems. These data were available until 31 December 2016 for women screened at 26 years of age or older, and until 31 December 2018 for women screened at 24–25 years of age. For all women with positive HR-HPV tests regardless of their age, follow-up histology diagnoses were available until December 2018. All diagnoses were reported as assigned by the routine services. It was assumed that tests were performed for primary screening if the laboratory had no record of an LBC or HR-HPV test within the previous 2 years and/or the test itself was not marked as a response to a recent abnormality. All other tests were excluded from the analysis. Women were also categorised according to the decile of the Index of Multiple Deprivation (IMD). The IMD is an area-based standard measure of deprivation in England (information on deprivation at the individual level is not routinely collected). In order to determine a woman’s IMD, her postcode at the time of screening was linked to the Lower Layer Super Output Area code in conjunction with the government’s English indices of deprivation report from 2015 [11]. A small number of women with an unknown IMD were excluded from the analysis.

### Patient and public involvement

Neither screened women nor the public was involved in the conduct of this study. A study to evaluate women’s psychological responses to HR-HPV testing was embedded in the pilot and its findings have been separately reported [12–14].

### Statistical analysis

The last of the six laboratories began reporting data to the pilot in August 2013, and women were included in the analysis if they were screened in September 2013 or later. We reported the annual proportions of women (with 95% exact binomial confidence intervals [CI]) with HR-HPV infections detected at baseline screening, HR-HPV infections in combination with non-negative cytology (a proxy for the number of referrals to colposcopy at baseline screening), diagnosis of CIN1, CIN2+, CIN3+ or cervical cancer, either squamous or glandular, after referral to colposcopy at baseline (for all referrals made until 30 June 2018, to give women time to attend the colposcopy), and the PPV of colposcopy for a CIN2+ diagnosis on histology. For the subset of the data from the four genotyping laboratories, we stratified these outcomes according to whether the infecting HR-HPV genotypes were those included in the vaccine (HPV 16/18) or not (12 “other” HR-HPV genotypes). Infections with “other” genotypes were counted independently of any co-infections with HPV 16/18; however, for the analyses of CIN2+ and CIN3+ associated with these “other” HR-HPV genotypes, cases of 16/18 co-infections were excluded, as the latter were the more likely drivers for the development of a CIN lesion. The PPV of colposcopy for CIN2+ was calculated as the proportion of women who underwent a colposcopy because of an HR-HPV-positive baseline test with non-negative triage cytology, with a diagnosis of CIN2+.

The annual trends in the proportions of screened women who had abnormalities detected by screening were tested using log-binomial regression using the glm command with binomial family and log link in Stata 15.0. These models included year as a continuous predictor. All models were adjusted for (a) IMD decile, because women from more deprived backgrounds are known to be more likely to develop CIN and cervical cancer [15, 16], and (b) laboratory, in order to take account of unmeasured local characteristics associated with screening outcomes, to obtain adjusted prevalence ratios (PR_adj_). Analyses were reported separately for women aged 24–25, 26–27, and 28–29 years at screening.

The observed time trends in screening abnormalities at the age of 24–25 years were compared with the increasing vaccination coverage in the population. Data on the individual vaccination status of women included in the pilot were not available for analysis. As before [17], the age and calendar-year specific probability that a woman was vaccinated was estimated from the official national statistics for vaccination with three doses in the general population, available by school cohort (Supplementary Information, Table S1). To estimate the vaccination coverage in the population of women undergoing screening, we calculated a weighted average of the assigned age and calendar-year specific probabilities for women aged 24 vs. 25 years who participated in screening in the pilot in each calendar year.

The reported time trends in screening outcomes describe the differences between a population to which vaccination had not been offered (earlier years), and a population with an increasing proportion of women who received the vaccination as part of the catch-up campaign and a decreasing proportion of women who were eligible for this vaccination but did not obtain it (later years). As the information on the individual women’s vaccination status was not available, we could not estimate the relative differences in screening outcomes between vaccinated and unvaccinated women (i.e. the vaccine effectiveness) directly. Instead, we used log-binomial regression models that included a continuous variable for the assigned vaccination coverage (as a proportion, see above) to estimate prevalence ratios [18] adjusted for IMD decile and laboratory. These PR_adj_ provided an estimate of the relative differences in the prevalence of screening abnormalities between a fully vaccinated population (100% vaccination coverage) and an unvaccinated population (0% vaccination coverage) for all years combined (2013–2018). The vaccine effect was then estimated by subtracting the PR_adj_ for the vaccination coverage from 1 (i.e. 1 − PR_adj_) to estimate the reduction in the risk due to vaccination for women who were screened at age 24–25 years. An example of this calculation is presented in the Supplementary Information. To test the robustness of this model, we separately estimated vaccine effectiveness by assuming that the proportion of vaccinated women among those screened was 20% higher than in the analysis described above (see Supplementary Information, Table S1, for absolute values).

Stata version 15 and RStudio version 1.1.463 were used for the analyses.

## Results

### Screening outcomes at the age of 24–25 years for birth cohorts that were offered vaccination

**Fig. 1:**
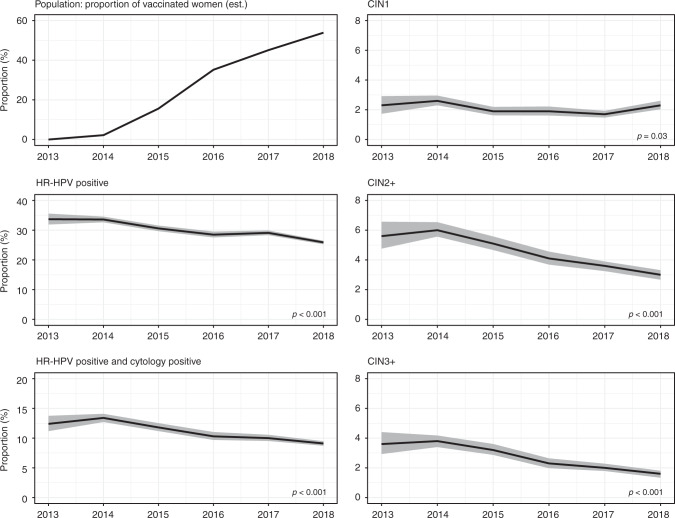
Time trends in screening outcomes in women aged 24–25 years and the proportion of vaccinated women (as estimated from population-based official statistics data). Grey areas: 95% confidence intervals for proportions. All reported values for screening outcomes were calculated as proportions per 100 screened women. CIN1, CIN2+, and CIN3+ denote lesions detected at baseline colposcopy after an HR-HPV-positive screening sample with abnormal cytology.

As part of the pilot, 64,274 women aged 24–25 years were screened using HR-HPV testing between September 2013 and December 2018 (Table 1). During this period, the proportion of women who reached this age and received three doses of the bivalent HPV vaccine increased from 0% to just under 55%, as estimated from population-based statistics (Fig. 1). These women became eligible for vaccination at ages 14–17 years, although for the majority this would be at ages 15–17 years (Supplementary Information, Table S1).

**Table 1:** Outcomes in women screened at the age of 24–25, by calendar year.

	2013	2014	2015	2016	2017	2018^a^	*p* for trend^b^	Vaccine effectiveness (95% CI)
Overall results (6 laboratories)								
* N* screened	2600	9486	9179	8157	12,949	21,903		
HR-HPV+	877 (33.7%)	3188 (33.6%)	2810 (30.6%)	2328 (28.5%)	3770 (29.1%)	5662 (25.9%)	<0.001	41% (36 to 45)
HR-HPV+ and cytology+	323 (12.4%)	1270 (13.4%)	1087 (11.8%)	844 (10.3%)	1299 (10.0%)	1992 (9.1%)	<0.001	49% (43 to 54)
CIN1^c^	59 (2.3%)	248 (2.6%)	174 (1.9%)	155 (1.9%)	219 (1.7%)	255 (2.3%)	0.03	28% (4 to 46)
CIN2+^c^	146 (5.6%)	573 (6.0%)	469 (5.1%)	335 (4.1%)	461 (3.6%)	329 (3.0%)	<0.001	72% (66 to 77)
CIN3+^c^	94 (3.6%)	358 (3.8%)	296 (3.2%)	187 (2.3%)	262 (2.0%)	172 (1.6%)	<0.001	79% (73 to 83)
Cervical cancer^c^	NR	NR	NR	NR	NR	NR	0.14	64% (−91 to 93)
Had colposcopy (PPV for CIN2+)	322 (45.3%)	1241 (46.2%)	1053 (44.5%)	790 (42.4%)	1202 (38.4%)	1046 (31.5%)		
Genotyped results (4 laboratories)								
*N* screened	1803	6836	6533	5734	8337	13,146		
HPV 16/18+	213 (11.8%)	923 (13.5%)	645 (9.9%)	381 (6.6%)	459 (5.5%)	407 (3.1%)	<0.001	90% (89 to 92)
HPV 16/18-related CIN2+^c^	59 (3.3%)	289 (4.2%)	216 (3.3%)	116 (2.0%)	145 (1.7%)	71 (1.2%)	<0.001	87% (82 to 91)
HPV 16/18-related CIN3+^c^	42 (2.3%)	193 (2.8%)	158 (2.4%)	75 (1.3%)	106 (1.3%)	45 (0.7%)	<0.001	87% (80 to 91)
“Other” HR-HPV+^c,d^	503 (27.9%)	1847 (27.0%)	1635 (25.0%)	1462 (25.5%)	2251 (27.0%)	3292 (25.0%)	0.06	1% (−7 to 8)
“Other” HR-HPV-related CIN2+^c,e^	35 (1.9%)	125 (1.8%)	126 (1.9%)	110 (1.9%)	143 (1.7%)	94 (1.5%)	0.01	34% (3 to 55)
“Other” HR-HPV-related CIN3+^c,e^	20 (1.1%)	68 (1.0%)	66 (1.0%)	57 (1.0%)	64 (0.8%)	37 (0.6%)	0.001	57% (25 to 75)

*CIN* cervical intraepithelial neoplasia, *HR-HPV* high-risk human papillomavirus, *NR* not reported due to small numbers in some of the years (total: 32), *PPV* positive predictive value of a colposcopy.

^a^For analysis of histological outcomes at colposcopy, only the first 6 months of the year was included in the analysis. In total, 11,042 women were screened with HR-HPV testing between January and June 2018 (of which 6168 in the 4 laboratories reported genotyped results).

^b^Adjusted for IMD and site.

^c^Detected after a HR-HPV+/cytology+ primary screening test that resulted in a referral to colposcopy at baseline.

^d^Includes any co-infections with HPV 16/18.

^e^Excludes CIN2+ and CIN3+ where co-infections with HPV 16/18 were present.

**Fig. 2:**
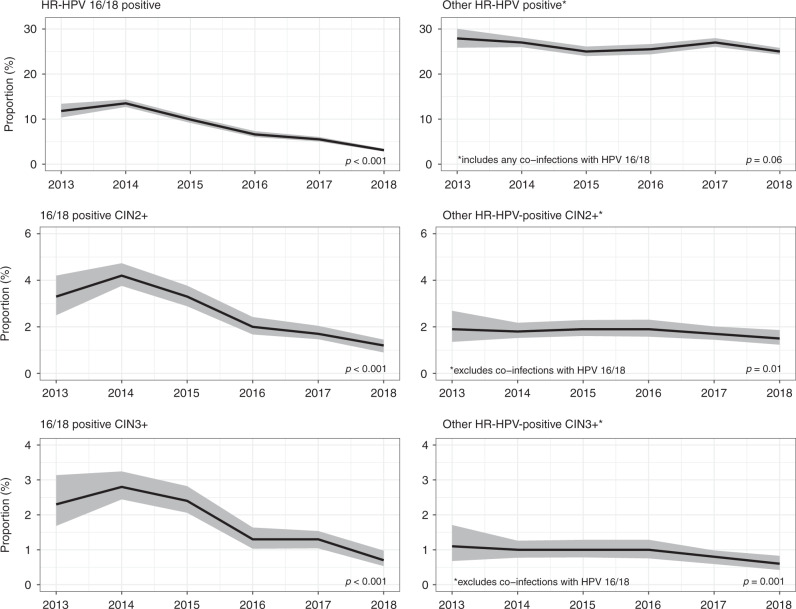
Time trends in HR-HPV genotyped screening outcomes in women aged 24–25 years (data available from 4 out of the 6 pilot laboratories). Grey areas: 95% confidence intervals for proportions. All reported values for screening outcomes were calculated as proportions per 100 screened women. CIN2+ and CIN3+ denote lesions detected at baseline colposcopy after an HR-HPV-positive screening sample with abnormal cytology.

In this age group, the proportion of screened women who had a positive HR-HPV test decreased from 34% in 2013–2014 to 26% in 2018 (Table 1 and Fig. 1; *p* for trend, adjusted for IMD and laboratory: <0.001). The decrease in the proportion was more pronounced for vaccine genotypes 16/18, where the prevalence in 2018 decreased by about three-quarters compared with the prevalence observed in 2013–2014 (from 13 to 3%; *p* < 0.001, Fig. 2). HPV genotypes 16 and 18 were less likely to occur in co-infections in cohorts offered vaccination: the proportion of HPV 16/18 infections accounted for almost 40% of all infections in 2013–2014 but fell to just over 10% by 2018 (data not tabulated). The extent of the decrease in overall HR-HPV positivity in the screening population applied similarly to both types 16 and 18 and was similar in those laboratories using DNA assays and those using mRNA assays (Supplementary Information, Figs. S2 and S3, respectively). The change in the population prevalence of the “other” HR-HPV genotypes was much less marked and the proportion with such infections oscillated between 25 and 27% (*p* = 0.06). The estimated effectiveness of the vaccine (i.e. the estimated relative difference between vaccinated and unvaccinated women) was around 40% against HR-HPV infections overall, and around 90% against HPV 16/18 infections (Table 1).

The proportion of women aged 24–25 years with a HR-HPV-positive screening test and abnormal triage cytology (i.e. those that were referred to colposcopy at baseline) decreased from 13% in 2013–2014 to 9% in 2018 (*p* < 0.001). The observed proportion of women with CIN1 among those screened oscillated around 2% (*p* = 0.03). The proportion of those with CIN2+ approximately halved from 6 to 3% (*p* < 0.001), as did the proportion with CIN3+, which halved from 4 to 2% (*p* < 0.001). The degree of protection exerted by the vaccine (i.e. the effectiveness) was estimated to be 70–80% for all high-grade CIN lesions (Table 1), while for CIN2+ and CIN3+ associated with HPV 16/18, the vaccine effectiveness was estimated at nearly 90%. For high-grade CIN associated with “other” HR-HPV genotypes, the decrease in the detection was smaller (e.g., CIN2+ associated with these genotypes continued to be detected in 1–2% of the screened population), and the estimated vaccine effectiveness was less than that seen for genotypes 16/18: about 30% for CIN2+ and 60% for CIN3+ (with broad 95% CIs).

Although the unadjusted observed detection of cervical cancer at screening decreased by about 75% between 2013 and 2018 (from >0.1% to <0.05%), the trend did not reach statistical significance. The *p* value was 0.14, based on 32 cases diagnosed in 2013–2018, and vaccine effectiveness was estimated to be 64% (95% confidence interval (CI): −91 to 93%).

**Fig. 3:**
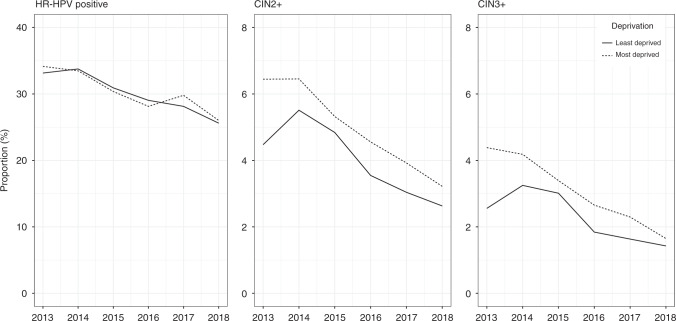
Time trends in overall HR-HPV positivity and CIN2+ and CIN3+ detected at baseline colposcopy after an HR-HPV-positive screening sample with abnormal cytology aged 24–25 years, by deprivation status. Least deprived: IMD deciles 6–10. Most deprived: IMD deciles 1–5. All reported values for screening outcomes were calculated as proportions per 100 screened women.

Figure 3 shows that at the age of 24–25 years, the observed time trends were similar for women with more vs. less deprived backgrounds (IMD deciles 1–5 vs. 6–10, respectively). The difference in HR-HPV positivity was small in birth cohorts that were not offered vaccination and remained small even in birth cohorts that were offered catch-up vaccination. Detection of CIN2+ and CIN3+ was higher in women from more deprived backgrounds throughout the observation period. In cohorts offered vaccination, however, the difference in the detection of high-grade CIN between more and less deprived backgrounds became smaller than it was in older cohorts.

**Fig. 4:**
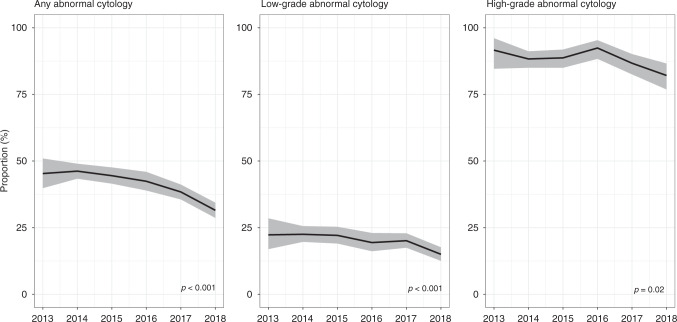
Time trends in the positive predictive value for CIN2+ at baseline colposcopy after an HR-HPV-positive screening sample with abnormal cytology in women aged 24–25 years. Grey areas: 95% confidence intervals for proportions. All reported values for screening outcomes were calculated as proportions per 100 screened women.

The PPV, for CIN2+, of a colposcopy after a positive HR-HPV primary screening test with non-negative triage cytology decreased from 46% in 2013–2014 to 32% in 2018 (Fig. 4). The decreasing trend was observed, particularly after year 2015, in both low-grade (*p* < 0.001) and high-grade (*p* = 0.02) cytological abnormalities.

Additional analyses assuming that the proportion of vaccinated women among those who were screened was 20% higher than estimated from the general population data produced slightly lower estimates of vaccine effectiveness (not tabulated). For prevention of HPV 16/18 infections, for example, the estimate decreased from 90% (95% CI: 89–92) to 86% (95% CI: 84–88); for prevention of CIN2+ associated with HPV 16/18 infections, it decreased from 87% (95% CI: 82–91) to 82% (95% CI: 76–86). Similarly, time trends adjusted for IMD and site without specifying any assumption on vaccine effectiveness exhibited, in general, highly statistically significant decreases in the proportions of women with screen-detected abnormalities.

### Screening outcomes at the age of 26–29 years for birth cohorts that were not offered vaccination

Between September 2013 and December 2016, 16,864 women aged 26–27 years had a primary screening test, as did 27,000 women aged 27–28 years (Supplementary Information, Tables S2 and S3). Practically none of these women had been offered HPV vaccination (Supplementary Information, Table S1).

**Fig. 5:**
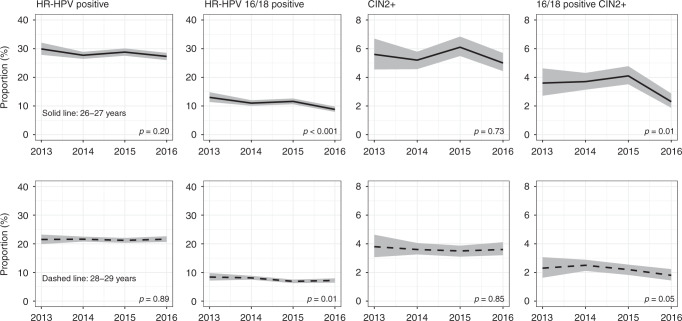
Time trends in screening outcomes in women aged 26–27 and 28–29 years. Grey areas: 95% confidence intervals for proportions. All reported values for screening outcomes were calculated as proportions per 100 screened women. CIN2+ denote lesions detected at baseline colposcopy after an HR-HPV-positive screening sample with abnormal cytology.

In both age groups, decreases in overall HR-HPV prevalence were small, if observed at all (*p* = 0.20 and *p* = 0.89, respectively; Fig. 5). Nevertheless, the prevalence of HPV 16/18 decreased by about one-quarter (from 12% in 2013–2014 to 9% in 2018, *p* < 0.001) in the age group 26–27 years and by about one-seventh (from 8 to 7%, *p* = 0.01) in the age group 28–29 years, while it decreased by three-quarters in the age group 24–25 years (as shown above). The prevalence of “other” 12 HR-HPV genotypes (in combination) remained stable throughout the observation period (at around 22%, *p* = 0.88, at age 26–27 years, and at around 17%, *p* = 0.96, at age 28–29 years).

The proportion with high-grade CIN associated with HPV 16/18 decreased by about one-third in the age group 26–27 years (*p* = 0.01 for both CIN2+ (from just <4% to just >2%) and CIN3+ (from 3 to 2%)). The decreases were less pronounced at 28–29 years of age (from just >2% to just <2%, *p* = 0.05, for CIN2+, and from around 2% to around 1%, *p* = 0.10, for CIN3+). At 26–27 years of age, there was a suggestion of an increase in CIN2+ (from 2 to 3%, *p* = 0.01) and CIN3+ (from 1 to 2%, *p* = 0.05) associated with “other” HR-HPV genotypes (using, as explained above, the definition of “other” infections that excluded co-infections with HPV 16/18).

## Discussion

### Principal findings

This large study of data from a pilot of routine primary screening with HR-HPV testing in England contributes to the body of international studies that show that HPV 16/18 vaccination significantly reduces the risk of high-grade CIN lesions and cervical cancer. The data cover the first 4 years of screening of birth cohorts that were eligible for bivalent Cervarix vaccination through a catch-up campaign at the start of the English HPV vaccination programme. As the average age at sexual debut in the UK is around 16 years, with about 30% of women having their first sexual intercourse before the age of 16 years [19], the “real-life” effectiveness of vaccination was expected to be somewhat less than the efficacy of close to 100% reported in female volunteers who were naive to vaccine genotypes when they entered randomised trials [20]. Nevertheless, the frequency of high-grade CIN diagnoses in the English CSP decreased significantly, with diagnoses related to HPV 16/18 decreasing by about two-thirds, we believe as a direct consequence of the vaccination programme. Overall, these trends implied a protective effect of approximately 90% from the bivalent vaccine administered at 14–17 years of age on high-grade CIN associated with HPV 16/18 infections, and of 30–60% on high-grade CIN associated with infections with “other” HR-HPV genotypes.

When interpreting the causal role of the vaccine in this ecological study, possible independent concurrent factors such as changes in sexual behaviour and background risk should be considered. Increasingly younger age at sexual initiation [21] and an increasing number of sexual partners [19] suggest that it is unlikely that the background risk of HR-HPV infections has decreased among younger birth cohorts. The CSP screening and colposcopy protocols were unchanged throughout the pilot in terms of the selection of women to be invited for screening, as well as the HR-HPV testing protocols and the clinical investigation of the detected infections. The national screening coverage of women aged 25–29 years remained virtually unchanged between 2013 and 2018 [22]. Indeed, the patterns observed in the pilot showed characteristics of dose-response relationships that would be expected for vaccination as a causal factor. The patterns showed specificity in that the decreases were most pronounced for vaccine genotypes. These decreases were first observed when the first cohorts that were offered vaccination entered the CSP and grew as cohorts with higher vaccination coverage and younger age at vaccination started entering the programme.

The ultimate goal of HPV vaccination is to prevent cervical cancer. Recently published data from Sweden confirmed a lower incidence of cervical cancer in women vaccinated with the quadrivalent vaccine [3], while cancer registry data from England confirmed a lower incidence of cervical cancer in women vaccinated with the bivalent vaccine [23]. Our data were consistent with these findings. We observed fewer cases of cancer detected after the first screening invitation at the age of 24–25 years, although our data from this initial period lacks power for cervical cancer outcomes.

### Strengths and limitations of the study

The greatest strength of these data is that they were derived from a prospective, large-scale, and real-life pilot study, fully embedded in a highly effective CSP with national quality assurance guidelines and monitoring. It involved successive, unselected women who attended the screening in different areas throughout England. The English CSP, within which this pilot was performed, is one of the few programmes that incorporates HR-HPV testing for primary screening from the age of 25 onwards, so the value of our data stands out in the global context.

Previous real-world studies have had to rely on samples from volunteers, from women seeking health care for other reasons, or from women undergoing cytological screening where HR-HPV infections were determined from archived samples, often using highly sensitive epidemiological HPV assays [5]. In contrast to these earlier studies, all HR-HPV tests in the pilot were performed using the same clinically validated assays that had been selected for the national programme. It can therefore be expected that these data offer reliable predictions for the CSP and other similar programmes.

The estimated vaccine effectiveness against high-grade CIN and HR-HPV infections must be interpreted with caution, as it was established in women who chose to undergo screening and we had no information about their individual vaccination status. Our estimates of vaccination coverage were taken from official national statistics for England. Early Scottish data from the catch-up campaign (from a screening programme that invited women for the first time at the age of 20 years) showed that, regardless of the level of deprivation, fully vaccinated women were more likely to be screened [24]. If the same pattern of screening participation was true in the six pilot areas in England, our regression analyses may have overestimated the differences between vaccinated and unvaccinated women. As shown in sensitivity analyses, however, the findings of high vaccine effectiveness remained robust.

Our study relied on routinely reported diagnoses and high-grade CIN histology was not tested to confirm aetiological associations with specific HR-HPV genotypes. By default, for women with genotyped results, we assumed that CIN was associated with “other” HR-HPV infections if no HPV 16/18 infections were detected in the same screening sample. The apparently increasing trend of high-grade CIN in cohorts not offered vaccination (at ages 26–27 years) may be a consequence of this definition in the context of changing epidemiology of multiple infections involving HPV 16/18, as co-infections of HPV 16/18 and “other” HR-HPV genotypes were more frequent before vaccination [25]. On the other hand, our study also provided an apparently inconsistent finding in cohorts offered vaccination (in our analysis, this refers to women aged 24–25 years), since the vaccine did not affect infections with “other” HR-HPV genotypes, but did have an effect on the high-grade CIN associated with these genotypes. This finding might be explained by cross-protection. Partial cross-protection by the vaccine has been well demonstrated for genotypes 31, 33, and 45 [26]. In unvaccinated populations, these three genotypes were among the genotypes most likely to produce high-grade CIN lesions, which can now be partly prevented by the vaccine [27], but overall they represented a small number compared with all other “other” HR-HPV infections, a majority of which are not prevented by the vaccine [25]. Genotype-specific cross-protection, however, could not be studied, as the available routinely reported HR-HPV test outcomes do not differentiate between the individual 12 “other” HR-HPV genotypes.

### Comparison with other studies

As far as the study designs can be compared, the patterns observed in the English pilot in cohorts that were offered vaccination between the ages of 14–17 years were consistent with those observed in previous studies. [5, 26, 28–32] In the PATRICIA randomised controlled trial, the efficacy of the bivalent vaccine against HPV 16/18-related high-grade CIN was close to 100% in women who were naive to HR-HPV before vaccination, but fell to 61% for CIN2+ and 46% for CIN3+ when all vaccinated women were included in the analysis [33]. The latter is lower than our estimates of around 90%, but in the trial, vaccination was administered between the ages of 15 and 25 years, with an average age of 20 years. Some degree of herd protection may have also played a part in making our estimates higher than were efficacy estimates from the trial. In the Scottish CSP, where until recently women received their first invitation for LBC screening at age 20 (as opposed to 24.5 in the English CSP) and individual vaccination status could be obtained from administrative records, it was estimated that bivalent catch-up vaccination administered at 14–17 years of age was about 70% effective against high-grade CIN [28].

Before our analysis, English data were only available from settings such as chlamydia screening or from sexual health clinics. There, the effect of the bivalent vaccine administered at 15–17 years of age against HPV 16/18 infections was estimated to be a 49% reduction [18]. Although this is lower than estimated in our analysis, women participating in chlamydia screening are probably not fully representative of the general population targeted by the CSP. They may have a higher risk of contracting an HR-HPV infection, an earlier age at sexual debut and lower-than-average vaccination uptake, instead of the likely higher-than-average uptake in women attending for cervical screening.

### Policy implications

These data indicate an impact of the HPV vaccine both beyond the vaccinated cohorts and beyond the vaccine genotypes. The decrease in high-grade CIN associated with non-vaccine genotypes indicated the presence of partial cross-protection against “other” HR-HPV genotypes, while a decrease in the prevalence of HPV 16/18 infections in older women not included in the catch-up campaign probably indicates some degree of herd protection to those older women. Both effects have already been observed in other routine settings [5, 26, 28]. It is expected that the screening of cohorts that were offered vaccination at the age of 12 or 13 years scheduled to begin in England in 2020, will have an added effect in reducing the incidence of cervical cancer precursor lesions [28]. This in turn will require a national reconsideration of screening target age ranges and intervals, leading to significantly fewer screening rounds during a woman’s lifetime in order to continue to deliver the CSP in a cost-effective manner [34]. More evidence on the safety of extended screening intervals is expected from the reporting of the Finnish randomised controlled trial in women who were vaccinated at either age 13–15 or 18 and thereafter screened in their twenties, where data from the baseline testing has already shown promising results [32].

Several years ago, concern was expressed about the use of cytology for screening vaccinated women, fearing that a decreasing prevalence of cervical lesions would lead to a deterioration in the PPV for CIN2+ [35]. Such decreases have subsequently been demonstrated in real-life settings, for example in Scotland and Sweden [36, 37]. Unlike in these two countries where LBC was used as the primary screening test, women in the English pilot were screened with HR-HPV testing, with LBC reserved for triage of HR-HPV-positive samples. Although the criteria for assessing LBC remained unchanged throughout the pilot, a decline in the PPV was also here observed in both low- and high-grade abnormal, HR-HPV-positive cytology. Among women who were referred to colposcopy in the pilot, the proportion of those with HPV 16/18 infections decreased over time as a consequence of vaccination; in parallel, the proportion referred with infections with “other” HR-HPV genotypes increased from around 50% to around 70% (not tabulated). Colposcopies in women with “other” HR-HPV infections had a lower PPV for CIN2+ (around 30% in women with concurrent abnormal cytology) than did colposcopies with HPV 16/18 infections (around 60%; not tabulated). This difference in the PPV between the two groups of genotypes was consistent with observations from Denmark, where the existing “other” HR-HPV infections, even if they persisted for several years, were less likely to lead to high-grade CIN than did HPV 16/18 [38]. Other potential factors such as differential ascertainment at colposcopy, e.g. due to a lower visibility of non-16/18 CIN lesions, may have also led to a lower PPV associated with “other” HR-HPV infections. In all, the changes in the PPV observed so far at 24–25 years of age, from 46 to 32%, mean that 3.1 (100/32) colposcopies were performed to detect a case of CIN2+ after (catch-up) vaccination, whereas in cohorts that were not offered vaccination this was 2.2 (100/46). The observed reductions in the number of women referred for colposcopy, and in the PPV of those colposcopies, will represent a highly significant change for colposcopy services as nearly half of all colposcopies in the CSP used to be performed in women under 30 years of age [8].

Other programme services that can be expected to be affected by vaccination are those related to cytological evaluation in the triage of HR-HPV infections. While the need for cytology capacity decreased by about 85% when the programme substituted LBC with HR-HPV testing [8], our data showed a further ~20% decrease in HR-HPV positivity at the age of 24–25 years by 2018. This decrease is expected to be even more pronounced once routinely vaccinated women enter the CSP.

Neither our data nor the data from Scotland [16] showed a widening of socioeconomic disparities in terms of the detection of cervical abnormalities at screening. Nevertheless, there are still a number of unvaccinated women, including those from socially disadvantaged backgrounds, who are also less likely to participate in screening [39, 40]. It should be noted that these women will have a lower absolute risk than prior to vaccination, due to partial herd protection [41]. Even higher levels of herd protection can be expected from the vaccination of males as well as females [42, 43], which several countries have adopted relatively recently, including England. Nevertheless, continued monitoring of the incidence of cervical cancer, which is already being carried out [2, 23], will remain crucial in assessing changes in relative social disparities.

In conclusion, our data provide further evidence that the impact of vaccination against HPV 16/18 delivered through a catch-up campaign is impressive. Within the English CSP, these findings add to the rationale for a review of the case for extended screening intervals for cohorts offered vaccination.

## Supplementary information

Appendix

Reproducibility Checklist
