# Supplementary material for: The impact of catch-up bivalent human papillomavirus vaccination on cervical screening outcomes: an observational study from the English HPV primary screening pilot
Source: Br J Cancer. 2022 Mar 26;127(2):278–87. doi: 10.1038/s41416-022-01791-w (PMC9296648; doi:10.1038/s41416-022-01791-w)
Supplement: Supplementary file 1 — Appendix [file 41416_2022_1791_MOESM1_ESM.docx]

Cancer Prevention Group

School of Cancer & Pharmaceutical Sciences

Faculty of Life Sciences & Medicine

King’s College London

London, 4 March 2022

**THE IMPACT OF CATCH-UP BIVALENT HUMAN PAPILLOMAVIRUS VACCINATION ON CERVICAL SCREENING OUTCOMES: AN OBSERVATIONAL STUDY FROM THE ENGLISH HPV PRIMARY SCREENING PILOT**

***SUPPLEMENTARY INFORMATION***

Matejka Rebolj, Francesca Pesola, Christopher Mathews,

David Mesher, Kate Soldan, Henry Kitchener,

on behalf of the English HPV Pilot Steering Committee

***************************

This document provides additional data breakdowns to support the findings from the main text.

Members of the HPV Pilot Steering Committee, other than those listed as authors, included (in alphabetical order): Tracey-Louise Appleyard, Margaret Cruikshank, Kate Cuschieri, Karin Denton, Kay Ellis, Chris Evans, Viki Frew, Thomas Giles, Alastair Gray, Miles Holbrook, Katherine Hunt, Tanya Levine, Emily McBride, Timothy Palmer, Janet Parker, Elizabeth Rimmer, Hazel Rudge Pickard, Alexandra Sargent, David Smith, John Smith, Ruth Stubbs, John Tidy, Xenia Tyler, Jo Waller.

Table S1. Estimated proportions of women in the general population who had received HPV vaccination (with three doses), by calendar year and age at screening. Numbers in brackets are ages at which women were offered HPV vaccination.

| **Age at screening** | **2013** | **2014** | **2015** | **2016** | **2017** | **2018** |
| --- | --- | --- | --- | --- | --- | --- |
| 24 | 0% | 7.9%  (17y) | 30.2%  (17y) | 43.3%  (16-17y) | 48.8%  (15-17y) | 64.1%  (14-16y) |
| 25 | 0% | 0% | 7.9%  (17y) | 30.2%  (17y) | 43.3%  (16-17y) | 48.8%  (15-17y) |
| 26 | 0% | 0% | 0% | 7.9%  (17y) | 30.2%  (17y) | 43.3%  (16-17y) |
| 27 | 0% | 0% | 0% | 0% | 7.9%  (17y) | 30.2%  (17y) |
| 28 | 0% | 0% | 0% | 0% | 0% | 7.9%  (17y) |
| 29 | 0% | 0% | 0% | 0% | 0% | 0% |

Shaded cells represent data available for analysis.

In a sensitivity analysis exploring the robustness of the estimates with regard to the vaccination coverage in the screened population, we re-ran some of the models assuming that the coverage was 20% higher than reported here. In that case, the following vaccination coverage estimates were used for women aged 24: 9.5% in 2014, 36.2% in 2015, 52.0% in 2016, 58.6% in 2017, and 76.9% in 2018. These values were applied also to women screened at age 25, as applicable.

Table S2. Outcomes in women screened at 26-27 years of age, by calendar year (baseline screening data available until end of December 2016).

|  | **2013** | **2014** | **2015** | **2016** | **P for trend (adj.)** |
| --- | --- | --- | --- | --- | --- |
| **Overall results (6 laboratories)** |  |  |  |  |  |
| N screened | 1837 | 5258 | 4984 | 4785 |  |
| HR-HPV+ | 550 (29.9%) | 1454 (27.7%) | 1435 (28.8%) | 1304 (27.3%) | 0.20 |
| CIN2+^a^ | 102 (5.6%) | 271 (5.2%) | 306 (6.1%) | 241 (5.0%) | 0.73 |
| CIN3+^a^ | 60 (3.3%) | 184 (3.5%) | 191 (3.8%) | 150 (3.1%) | 0.65 |
| PPV of colposcopy for CIN2+ | 46.4% | 48.4% | 52.5% | 47.6% | 0.71 |
| **Genotyped results (4 laboratories)** |  |  |  |  |  |
| N screened | 1564 | 4175 | 3988 | 3730 |  |
| HPV 16/18+ | 204 (13.0%) | 460 (11.0%) | 462 (11.6%) | 330 (8.8%) | <0.001 |
| HPV 16/18-related CIN2+^a^ | 56 (3.6%) | 154 (3.7%) | 164 (4.1%) | 87 (2.3%) | 0.01 |
| HPV 16/18-related CIN3+^a^ | 39 (2.5%) | 111 (2.7%) | 118 (3.0%) | 59 (1.6%) | 0.01 |
| “Other” HR-HPV+^b^ | 360 (23.0%) | 898 (21.5%) | 890 (22.3%) | 828 (22.2%) | 0.88 |
| “Other” HR-HPV-related CIN2+^a^ | 25 (1.6%) | 77 (1.8%) | 91 (2.3%) | 98 (2.6%) | 0.01 |
| “Other” HR-HPV-related CIN3+^a^ | 13 (0.8%) | 49 (1.2%) | 42 (1.1%) | 58 (1.6%) | 0.05 |

Abbreviations: CIN=cervical intraepithelial neoplasia. HR-HPV=high-risk human papillomavirus.

^a^ Detected after a HR-HPV+/cytology+ primary screening test that resulted in a referral to colposcopy at baseline.

^b^ Includes any co-infections with HPV 16/18.

Table S3. Outcomes in women screened at 28-29 years of age, by calendar year (baseline screening data available until end of December 2016).

|  | **2013** | **2014** | **2015** | **2016** | **P for trend (adj.)** |
| --- | --- | --- | --- | --- | --- |
| **Overall results (6 laboratories)** |  |  |  |  |  |
| N screened | 2425 | 8765 | 8991 | 6819 |  |
| HR-HPV+ | 522 (21.5%) | 1895 (21.6%) | 1905 (21.2%) | 1475 (21.6%) | 0.89 |
| CIN2+^a^ | 92 (3.8%) | 319 (3.6%) | 312 (3.5%) | 248 (3.6%) | 0.85 |
| CIN3+^a^ | 56 (2.3%) | 213 (2.4%) | 197 (2.2%) | 145 (2.1%) | 0.34 |
| PPV of colposcopy for CIN2+ | 50.5% | 47.5% | 46.3% | 49.1% | 0.91 |
| **Genotyped results (4 laboratories)** |  |  |  |  |  |
| N screened | 1807 | 6382 | 6604 | 4649 |  |
| HPV 16/18+ | 152 (8.4%) | 519 (8.1%) | 454 (6.9%) | 333 (7.2%) | 0.01 |
| HPV 16/18-related CIN2+^a^ | 41 (2.3%) | 158 (2.5%) | 143 (2.2%) | 84 (1.8%) | 0.05 |
| HPV 16/18-related CIN3+^a^ | 29 (1.6%) | 111 (1.7%) | 103 (1.6%) | 59 (1.3%) | 0.10 |
| “Other” HR-HPV+^b^ | 310 (17.2%) | 1046 (16.4%) | 1079 (16.3%) | 777 (16.7%) | 0.96 |
| “Other” HR-HPV-related CIN2+^a^ | 28 (1.5%) | 78 (1.2%) | 90 (1.4%) | 76 (1.6%) | 0.24 |
| “Other” HR-HPV-related CIN3+^a^ | 12 (0.7%) | 47 (0.7%) | 48 (0.7%) | 33 (0.7%) | 0.92 |

Abbreviations: CIN=cervical intraepithelial neoplasia. HR-HPV=high-risk human papillomavirus.

^a^ Detected after a HR-HPV+/cytology+ primary screening test that resulted in a referral to colposcopy at baseline.

^b^ Includes any co-infections with HPV 16/18.

Figure S1. Lexis diagram for the study: birth cohorts invited for HPV vaccination and HR-HPV based cervical screening in the CSP pilot.


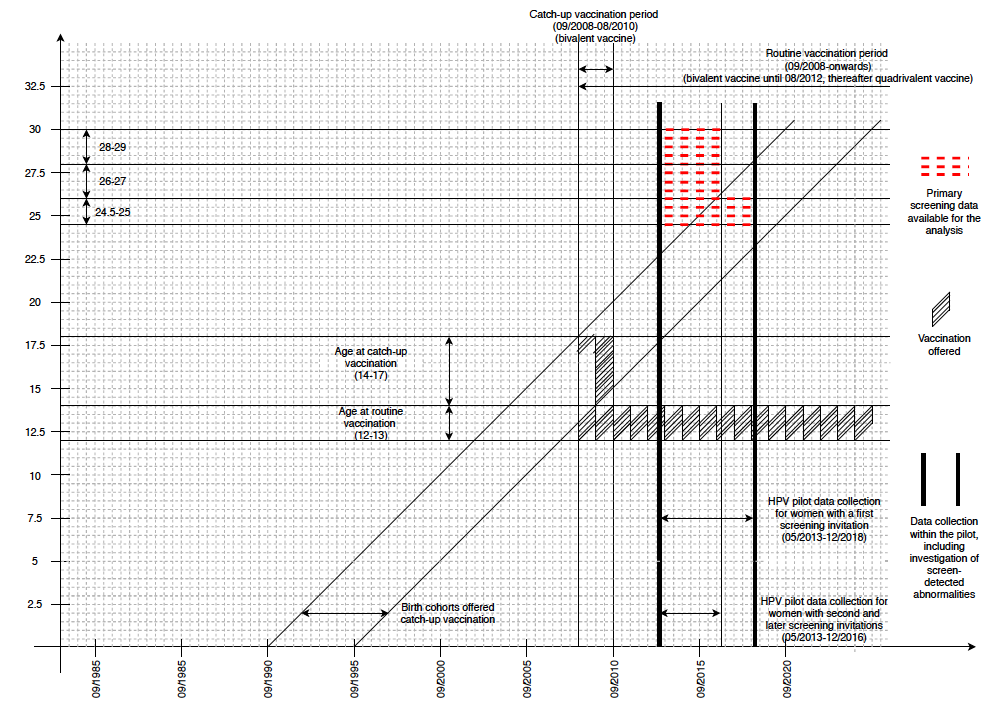


Figure S2. Time trends in the detection of HR-HPV infections in women aged 24-25 years in DNA vs. mRNA HR-HPV detection laboratories.

Grey areas: 95% confidence intervals for proportions.

Figure S3. Time trends in the detection of infections with HPV 16 and HPV 18 in women aged 24-25 years.

Grey areas: 95% confidence intervals for proportions.

**ADDITIONAL INFORMATION ON THE MODEL TO ESTIMATE VACCINE EFFECTIVENESS**

The log-binomial regression model to estimate vaccine effectiveness in women screened at age 24-25 years had the following form:

$$\log\left( p \right)= \sum_{i=0}^{j} \text{β}_{\text{i}}*\boldsymbol{x}_{\text{i}}$$

where *p* denotes the studied outcomes (various definitions of HPV prevalence and proportions of women with a detected CIN), while *x_i_* denotes a vector of explanatory variables: assigned vaccination coverage (as a proportion), decile of Index of Multiple Deprivation, and laboratory site.

Example

Outcome: proportion of women with an HPV16/18 infection.

Model output (using STATA):

Interpretation: The adjusted prevalence ratio for the vaccination effect (“vacc_cov”) was 0.10 (95% CI: 0.08-0.11) and, therefore, the protective vaccination effect was estimated to be (1-0.10)=0.90 (95% CI: 0.89-0.92).
